# Aqueous Extracts of Organic Mulch Materials Have Nematicide and Repellent Effect on *Meloidogyne incognita* Infective Juveniles: A Laboratory Study

**DOI:** 10.2478/jofnem-2023-0037

**Published:** 2023-09-01

**Authors:** Renáta Petrikovszki, Ferenc Tóth, Péter I Nagy

**Affiliations:** Department of Zoology and Ecology, Institute for Wildlife Management and Nature Conservation, Hungarian University of Agriculture and Life Sciences, H-2100, Páter Károly u. 1., Gödöllő, Hungary; Research Institute of Organic Agriculture, H-1033, Miklós tér 1., Budapest, Hungary.

**Keywords:** area choice, behavior, biological control, compost, leaf litter mulch, mortality, pH, tannic acid, wheat straw

## Abstract

While the nematicidal effectiveness of mulching against root-knot nematodes (*Meloidogyne* spp.) is calculated within organic crop protection, underlying mechanisms are not yet fully explored. Laboratory experiments were set up to determine whether mulch-derived substances cause mortality directly, or repel *Meloidogyne* juveniles from crop rhizosphere. Mortality and area choice tests were conducted with mulch-derived extracts, supported by the measurements on tannic acid content and the pH values of extracts as supplementary examinations. In our study, leaf litter and straw extracts were generally found lethal to the juveniles, which is in line with the results from area preference tests. However, compost extract had no effect on *Meloidogyne incognita* juveniles. Tannic acid content showed positive correlation with mortality only in the case of straw and sycamore leaf litter extracts. Tannic acid and pH weakly correlated with repellent effect of the applied extracts generally. Our results have inspired further experiments to explore nematicidal components of leaf litters, contributing to the development of a new approach in crop protection based on the repellent effect of these materials.

Plant-parasitic nematodes can cause losses of an estimated $100 billion worldwide, largely attributable to the damage caused by root-knot nematodes (*Meloidogyne* spp.) (Ralmi et al., 2016). *Meloidogyne* species have a wide range of host plants (Jones et al., 2013). In Hungary, their damages occur mainly on the roots of vegetables, weeds, and protected plant species (Dabaj et al., 1994; Bíró-Stingli and Tóth, 2011; Pinheiro et al., 2015). The symptoms could be manifested by various disorders in water and nutrient uptake and translocation, yield loss, dieback, yellowing, wilting of the canopy and the whole plant. They have been shown to colonize the root system of plants and cause galls or knots on the plant roots by the formation of giant cells due to the feeding of the second-stage juveniles of *Meloidogyne* (Sankaranarayanan and Hari, 2013; Khan et al., 2014).

Protection of crops against plant-parasitic nematodes is difficult because their eradication from the field is not sustainable, hardly even feasible (Khan et al., 2014). Pesticides, specifically nematicides, are effective and form the most common means of short-term management against most plant-parasitic nematode species (Hajihassani et al., 2019; Medina-Canales et al., 2019). In general, the use of nematicides on high-yielding crops has high returns, but the chemical control is sometimes not profitable for low-value crops (Haydock et al., 2006). In some regions, farmers lack the technical skills for the application of nematicides (Adediran et al., 2005). Re-treatments will be necessary year to year, especially if crops grown on the infested field are susceptible to plant-parasitic nematodes (Haydock et al., 2006). Only a few chemicals have been approved and registered as new nematicides recently, such as fluazaindolizine, fluopyram, and fluensulfone (Phani et al., 2021). However, certain nematicides (for example, fluensulfone) could be toxic for plants (Giannakou and Panopoulou, 2019), could accumulate along the food web, could be poisonous to animals and humans (oxamyl), or may enhance the depletion of the ozone layer (methyl bromide) (Hussain et al., 2017). An additional problem with using chemicals effectively is that certain plant-parasitic nematode species produce several generations within a year (Haydock et al., 2006).

On the other hand, granular or liquid formulations of soil fumigants are usually applied against plant-parasitic nematodes. The advantage of soil fumigants is that they do not have phytotoxic effects (Haydock et al., 2006) and they are more effective than nonfumigant contact or systemic nematicides, since they have a wide activity spectrum and can inactivate plant-parasitic nematodes for a longer time (Sikora et al., 2005). Therefore, the use of soil sterilization was decreased and alternative methods, such as biological control, were advised by the European Directive (2009/128/EK) (Williamson and Roberts, 2009). Due to the decreasing number of available chemical pesticides, the use of environmentally friendly and biological methods has been encouraged.

Various plant-derived extracts could be used as biopesticides (Godlewska et al., 2021; Nxumalo et al., 2021). Certain plants have developed physical or chemical mechanisms in order to protect themselves against pests and pathogens. These chemical compounds (e.g., phenols, poly-phenols, terpenoids, alkaloids) can be extracted from the plant by various methods, from simple aqueous soaking to the use of organic solvents to distillation (Dubey, 2011; Suteu et al., 2020). These extracts may have antifungal, antimicrobial activity against plant pathogens (Šernaitė, 2017) and a number of mechanisms have been developed against plant pests. These modes of action may include feeding inhibition, physical repellent, paralysis of nervous system, or inhibition of cellular respiration (Pavela, 2016; Suteu et al., 2020).

Lemon peel extract, aqueous extracts of Sodom apple (*Calotropis procera* (Aiton) Dryand) and castor (*Ricinus communis* L.), and higher-rate extract of neem (*Azadirachta indica* A. Juss.) tree inhibited the hatching of *Meloidogyne* eggs (Sharma and Trivedi, 2002; Bharadwaj and Sharma, 2007; Osei et al., 2011). The nematicidal activity of various parts of neem tree has been demonstrated in relation to several species of plant-parasitic nematodes (Akhtar, 2000). In addition, Azadirachtin, one of the active ingredients of neem, is also commercially available as an insecticide, which has a controversial effect on *Meloidogyne* species. Azadirachtin compound in pure form did not increase the immobilization and the mortality of *Meloidogyne* juveniles (Khanna and Kumar, 2006; Javed et al., 2007; Ntalli et al., 2009). On the other hand, ovicidal effect, immobility, and mortality of juvenile individuals were also observed when *Meloidogyne javanica* Treub eggs were exposed to aqueous extracts of neem leaves and cake (Javed et al., 2008). Maleita et al. (2017) examined extracts from the peel of black walnut (*Juglans nigra* L.) on *Meloidogyne hispanica* Hirschmann J2 individuals with an exposure time of 72 hours, during which period the examined extract caused a mortality of 82%. Ogwulumba et al. (2011) observed a very high nematicidal effect of aqueous extracts of bitter leaf (*Vernonia amygdalina* Delile) and mango (*Mangifera indica* L.) leaves. An extract of the fern *Dryopteris crassirhizoma* Nakai resulted in 100% mortality in *Meloidogyne* juveniles after 72 hours (Liu et al., 2013).

To summarize, these extracts may also be derived from parts of herbs used in human medicine, cultivated crops, or ornamental plants (Pavela, 2016; Suteu et al., 2020; Godlewska et al., 2021). In addition, compounds released from organic mulching materials may affect not only plant pests but also non-target organisms (Akhtar and Alam, 1992; Anderson, 2005).

Many of the cultivated plants or their extracts are used as soil amendments or for plant protection purposes worldwide. On the other hand, leaf litter, which becomes available every autumn in large amounts, has not received sufficient attention from this aspect yet, even though leaves from certain plants have a nematicidal effect. At the same time, underlying mechanisms, especially repellent or attractant modes of actions, are not completely understood yet (Spiegel et al., 2005; Sobkowiak et al., 2018). Therefore, experiments were set up under laboratory conditions to investigate whether substances leaching from mulch materials cause a direct mortality, or whether these extracts repel *Meloidogyne incognita* juveniles from the rhizosphere.

In addition to the laboratory tests, we also aimed to quantitatively identify a compound that can be found in all of the extracts included in this current study. Therefore, tannic acid was chosen, which is a hydrolysable tannin, a type of polyphenol (Chung et al., 1998).

## Materials and Methods

### Preparation of agar medium

Ten grams of agar was dissolved in 500 ml of distilled water and autoclaved. Since agar substrate is hot in liquid state, water from condensation appears on the inner side of the lid of the Petri dish. This condensation water can drop down to the surface of agar, which may cause nematode locomotor difficulties. Therefore, to obtain a dry surface agar, the lids of the Petri dishes were put on the bottom, with a 5 mm-wide gap to avoid the condensation water on the agar surface and to allow for evaporation. Petri dishes were left for 15 hours in a sterile laminar box. UV light was switched on for 30 minutes before the use of agar.

### Preparation of mulch-derived extracts

The following mulch materials were used: leaf litters from common walnut (*Juglans regia* L.), Norway maple (*Acer platanoides* L.), sycamore (*Platanus* × *hybrida* Brot.), common oak (*Quercus robur* L.), green yard waste compost (‘Zöld Híd Komposzt’ 04.2/3245-2/2017 Nébih), and straw of common wheat (*Triticum aestivum* L.).

Collected mulch materials were dried at 25 °C and 20% relative humidity for 2 days. 2.5 g of each mulch material was ground in a coffee grinder (Bosch MKM 6000) for 15 seconds.

Subsequently, 50 ml of high-purity water (Milli-Q) was added to the powder, mixed carefully, covered with aluminum foil, and soaked at room temperature. After 24 hours, the stock solutions (5% w/v) were filtered through cotton wool and additional concentrations of 1; 0.5 and 0.1% were diluted by adding Milli-Q water.

### *Meloidogyne incognita* culture

Egg masses of *Meloidogyne incognita* (Kofoid and White) Chitwood were collected from tomato roots (*Solanum lycopersicum* L. “Dány”). Egg masses were suspended in tap water and kept at 24 ± 1 °C for hatching. After 7 days, hatched second-stage juveniles were checked under a dissecting microscope with transmitting illumination (Olympus SZH 10) at a 30× magnification. Only viable *M. incognita* juveniles were used in the experiments.

### Mortality test with mulch-derived extracts

Mortality tests were performed in flat-bottom 96-well microplates (Kartell S.p.A., Italy) with the same methodology as in Petrikovszki et al. (2019). Individuals of *M. incognita* juveniles were sucked up one by one with an automatic pipette (capacity of 20–200 μL), and a total of 5 individuals, with the 20 μL water, were placed into each well. In this way, we were able to eliminate non-viable individuals, which could have biased the results. Milli-Q water served as control with 8 replicates; 4 replicates/concentrations were used for each extract and the test were repeated two times. Nematode viability was checked after 24 hours under a transmission stereomicroscope and juveniles were agitated to move by dropping 5% of lactic acid (modified method of 4% lactic acid by Ciancio, 1995) in order to check whether juveniles were immobile or actually dead. If mortality did not exceed 20% in the 0% control treatment, experiment was declared valid (Kiss et al. 2018).

### Area choice test with mulch-derived extracts

The setup was based on the combined and modified version of the method described by Hewlett et al. (1997) and Zhai et al. (2018): making holes/wells in the agar came from Hewlett et al. (1997), creating sectors to identify juveniles, and the exposure time (8 hours) originated from Zhai et al. (2018). However, a different design for the arrangement of holes and sectors, and 10% water agar in 6 cm diameter Petri dishes, were used. Then two holes, each 5 mm diameter, were cut into the agar plate. One hole served as the control, into which 50 μl of Milli-Q water was pipetted, while the hole on the opposite side always contained the mulch-derived extract to be tested, in a concentration of 5%, also in a volume of 50 μl.

Treatment pairs within a Petri dish were the following:

- MQ-water – MQ-water (control)
- Maple leaf litter extract – MQ-water
- Oak leaf litter extract – MQ-water
- Sycamore leaf litter extract – MQ-water
- Walnut leaf litter extract – MQ-water
- Straw extract – MQ-water
- Compost extract – MQ-water

Then 20–30 individuals of *M. incognita* juveniles were added with 20 μl water to the center of the Petri dish. Petri dishes were randomized on a tray and incubated for 8 hours in a thermostat (20 °C ± 1 °C) in darkness. Treatment pairs were replicated ten times, and the whole setup was done two times. After 8 hours, the number of juveniles on both sides were counted under a dissecting microscope with transmitting illumination (Olympus SZH 10) on 30× magnification.

**Figure 1:**
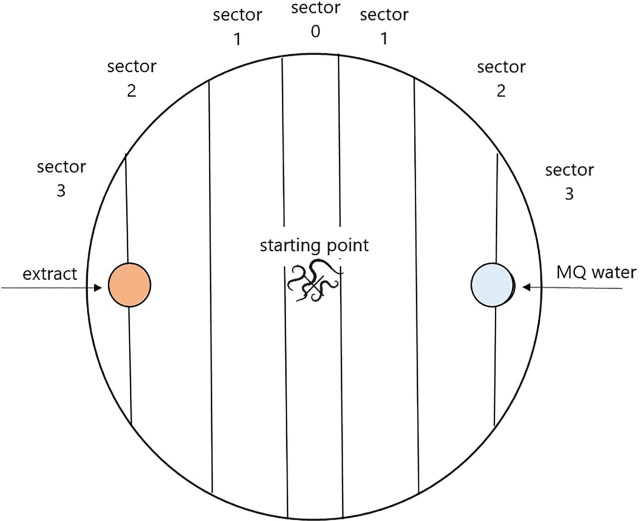
Representation of the sectors identified during the reading of the area choice test.

The sectors of the agar plate were drawn previously on a foil. The foil was placed under the Petri dish in order to determine the position of the juveniles (Figure 1). The number of juveniles found in the three sectors on the extract side was added up, so that the control side was counted in a similar way, and then the ratio between the two sides was given as a percentage. Juvenile individuals in ‘sector 0’ were not included on either side.

### Determination of tannic acid content of mulch-derived extracts

Determination of the tannic acid content of the mulch-derived extracts in our experiments followed the Hungarian Standard protocol for tannic acid determination by spectrometer ‘MSZ ISO 9648’ and was carried out by Corvinus Fitolabor Kft. of the Hungarian University of Agricultural and Life Sciences (Budapest, Hungary). Tannic acid was extracted by shaking with dimethylformamide. After centrifugation, iron (III), ammonium citrate, and ammonia were added to an aliquot part of the supernatant, then the absorbance of the solution was measured by spectrometric determination at 525 nm. Using tannic acid, a calibration curve was prepared, based on the determined tannic acid content. Tannic acid content of mulch-derived extracts was measured as mg/ml.

### Determination of pH values of mulch-derived extracts

The pH measurements were repeated five times by Voltcraft pH-212 device. Before measuring, pH meter calibration was performed by the two-point standardization method (pH 7 and pH 4) as described in the instructions.

### Statistical analysis

Mortality data of percentage values were square root arcsine-transformed in MS Excel 2016 before being analyzed by PAST3 (Hammer et al., 2001). One-way ANOVA, pairwise Tukey test, or Mann–Whitney U test (depending on the normality of data according to Shapiro-Wilk test) was used to analyze the average mortality values caused by various concentrations of each mulch-derived extract, in the case of mortality tests.

In area choice tests, percentage values of individuals of treatment pairs within Petri dishes were square root arcsine-transformed in MS Excel 2016 before Independent Samples T-test. *P* ≤ 0.05 level of significance was determined for every statistical analysis.

For linear regression, analyses were made to reveal the relationship between the pH value or tannic acid content as independent factors and the mortality of *M. incognita* juveniles as a dependent factor. In addition, in the case of area choice test, pH values served as an independent factor and the percentage of avoidance of *M. incognita* larvae as a dependent factor.

## Results

### Mortality test

**Figure 2:**
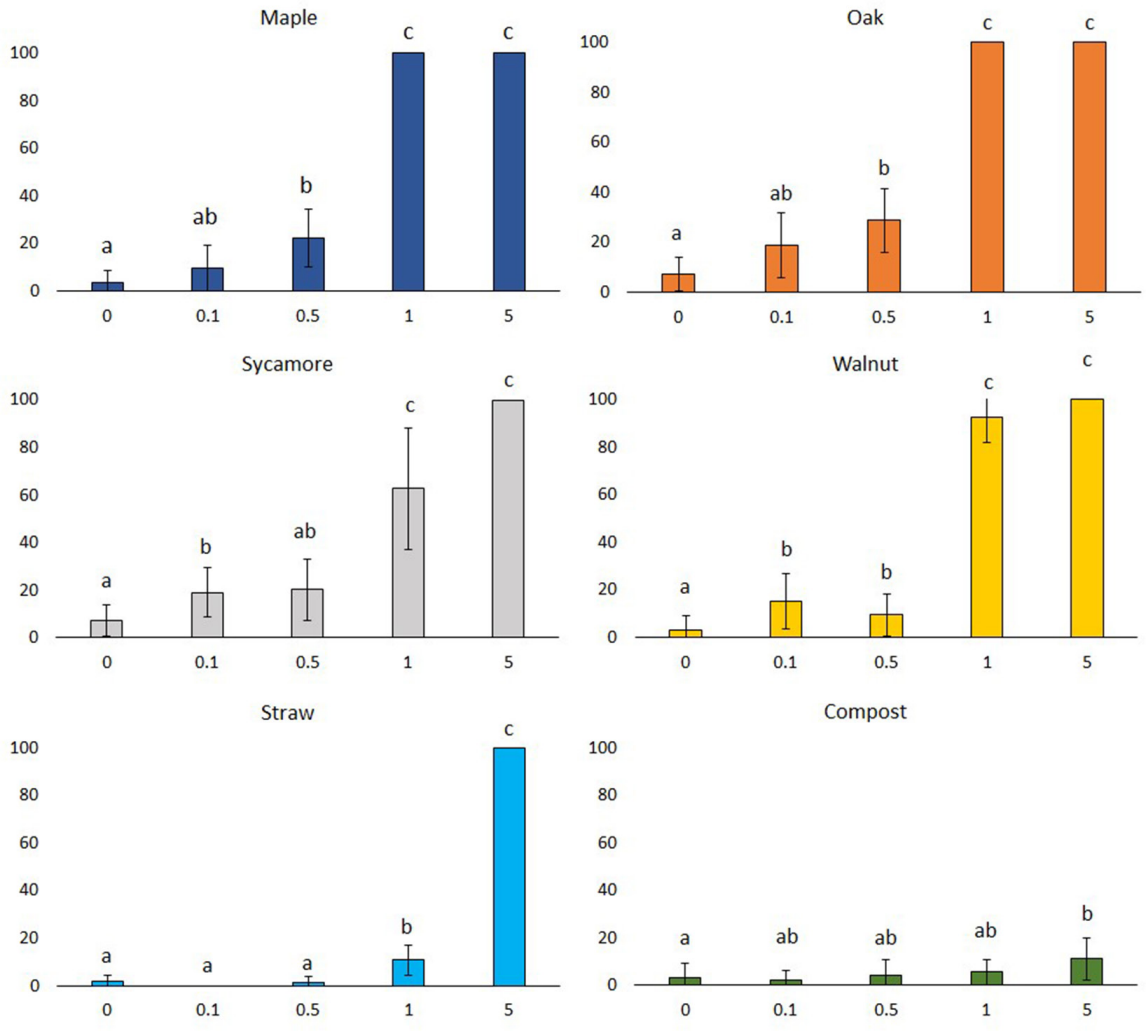
Mortality rates (mean ± CI 95%) of *Meloidogyne incognita* juveniles of different concen-trations (0.1, 0.5, 1 and 5%) of various leaf litter, straw and compost extracts. (CI 95%: 95% of confidence interval) (One-way ANOVA, Tukey's pairwise comparisons (in the case of compost), Mann–Whitney U test; different letters in the same row indicate significant difference at *p* ≤ 0.05 level).

A steep rise started in 0.5% of maple and oak leaf litter extracts, and then reached a peak at concentrations of 1%, which caused 100% mortality. Walnut leaf litter extract showed lower values at concentrations of 0.1 to 1% as compared to the previously mentioned extracts. At 0.5% concentration, mortality hit a slightly lower value, then increased and remained constant at the level of 100% mortality. In the case of sycamore leaf litter extract, there was a slight fall at 0.5% concentration, then rose to 29.6% at 1% concentration and reached the top of the mortality value at the highest concentration. In the lower (0.1, 0.5, and 1%) concentrations of straw extract, mortality remained low as well. However, in the 5% concentration, mortality value soared and peaked at 100%. As a contrast, compost extracts showed very low mortality values at each concentration evaluated or examined (Figure 2).

### Area choice test

**Figure 3:**
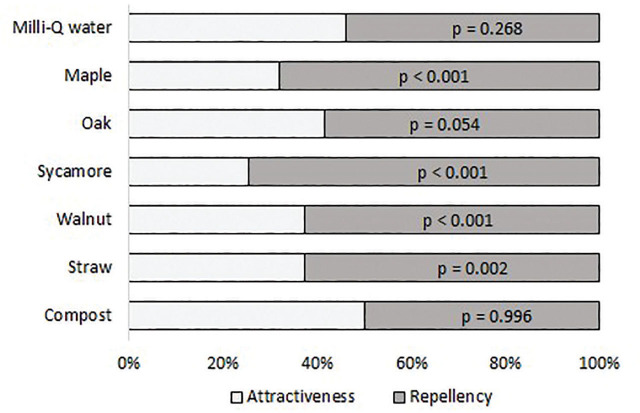
Area choice of *Meloidogyne incognita* juveniles with various leaf litter, straw and compost extracts (treated) and MQ-water (control). (Welch-test, *p* ≤ 0.05, *p*-value means that the number of *M. incognita* juveniles counted on the treated side is significantly different from that on the control side).

In the area choice study of *M. incognita* juveniles, both of the sides of the Milli-Q water control were selected in almost equal percentages (46%–54%). No difference was observed between the compost extract and the Milli-Q water treatment pairs (50%–50%) either. However, *M. incognita* juveniles avoided the sides treated with leaf litter extracts in the following percentage relative to the MQ water side: oak leaf litter 59%, walnut leaf litter 63%, maple leaf litter 68%, sycamore leaf litter 75%. Straw extract, similarly to walnut leaf litter, repelled 63% of the juveniles (Figure 3).

### Determination of tannic acid content and pH value of mulch derived-extracts

Among the extracts used in the experiments, the tannic acid content of the maple leaf litter extract was the highest, which was followed by the extract of oak leaf litter, sycamore leaf litter, walnut leaf litter, and finally straw extract. No tannic acid content could be measured from the compost extract (Table 1).

**Table 1.** Tannic acid content of different concentrations of various leaf litter, straw, and compost extracts.

**Tannic acid content (%)**					

**Concentrations (%)**	**0**	**0.1**	**0.5**	**1**	**5**

**Extracts**					
Maple leaf litter	n.d.	0.00521	0.02605	0.0521	0.2605
Oak leaf litter	n.d.	0.00241	0.01205	0.0241	0.1205
Sycamore leaf litter	n.d.	0.00209	0.01045	0.0209	0.1045
Walnut leaf litter	n.d.	0.0008	0.004	0.008	0.04
Straw	n.d.	0.00002	0.0001	0.0002	0.001
Compost	n.d.	n.d.	n.d.	n.d.	n.d.

(n.d.: non detectable)

The pH of the concentrations of 5% of the tested leaf litter and straw extracts fell into the slightly acidic category (except compost extract). Within this, the most acidic value was drawn from the 5% concentration of maple leaf litter, then oak leaf litter, sycamore leaf litter, walnut leaf litter, and finally straw extract. The compost extract was slightly alkaline, with a pH value of 7.79 (Table 2).

**Table 2.** Values (mean ± CI 95%) of pH of different concentrations of various leaf litter, straw, and compost extracts. (CI 95%: 95% of confidence interval).

**pH value**					

**Concentrations (%)**	**0**	**0.1**	**0.5**	**1**	**5**

**Extracts**					
Maple leaf litter	7.02 ± 0.04	5.33 ± 0.01	4.81 ± 0.02	4.78 ± 0.03	4.43 ± 0.03
Oak leaf litter	7.02 ± 0.04	5.95 ± 0.01	5.42 ± 0.02	5.23 ± 0.03	4.99 ± 0.03
Sycamore leaf litter	7.02 ± 0.04	6.42 ± 0.01	6.09 ± 0.03	5.36 ± 0.03	5.12 ± 0.02
Walnut leaf litter	7.02 ± 0.04	6.30 ± 0.01	6.63 ± 0.03	6.68 ± 0.03	6.21 ± 0.04
Straw	7.02 ± 0.04	6.37 ± 0.02	6.41 ± 0.04	6.44 ± 0.01	6.46 ± 0.03
Compost	7.02 ± 0.04	6.90 ± 0.01	7.29 ± 0.03	7.57 ± 0.03	7.79 ± 0.06

Based on the values of the regression analysis, the pH value of the oak leaf litter extracts was strongly correlated (r = 0.854) with the mortality values. The lethal effects of sycamore leaf litter extracts were increased by concentration (r = 0.919) and tannic acid content (r = 0.919). There is a similar trend for straw extract: concentration (r = 0.982) and tannic acid content (r = 0.982). In the case of compost extracts, there is no strong correlation with mortality for any of the parameters studied (Table 3).

**Table 3.** Correlation between the concentration, the tannic acid content, the pH of mulch-derived extracts and the percentage mortality of the juveniles of *Meloidogyne incognita*.

**Extracts**	**Concentration/Mortality**	**Tannic acid/Mortality**	**pH/Mortality**
Maple leaf litter	0.732	0.732	0.785
Oak leaf litter	0.710	0.710	0.854
Sycamore leaf litter	0.919	0.919	0.766
Walnut leaf litter	0.718	0.718	0.495
Straw	0.982	0.982	0.267
Compost	0.522	0.108	0.495

According to further regression analysis, area preferences of *M. incognita* juveniles were slightly influenced by pH (r = 0.387) and tannic acid content (r = 0.302) of mulch-derived extracts.

## Discussion

In the case of 20% concentration of common walnut leaf litter extracts, 100% mortality of *M. javanica* juveniles was observed after a 72-hour exposure (Fekrat et al., 2016). As a contrast in our experiment, the same result was seen in the presence of 1% of common walnut leaf litter extract after one day. There can be two reasons behind the different results. Firstly, different *Meloidogyne* species were used: in the cited experiment (Fekrat et al., 2016), *M. javanica* was applied, while our test species was *M. incognita.* These two species may show different sensitivity to phytochemicals. On the other hand, we prepared the extract from fallen leaves, while Fekrat et al. (2016) did not mention the exact origin of walnut leaves.

Yard waste compost (4-6-month-old) from sticks, clippings, and wood fragments did not have any nematicidal effect on *Meloidogyne* species in open-field experiments with several vegetable crops. The final population of *Meloidogyne* did not reduce or increase compared to the control area during experimental time (4–4.5 months) (McSorley and Gallaher, 1995). McSorley and Gallaher (1995) concluded that compounds leached from compost material may need more time to exert their influence, or their concentration may be too low to have any effect on nematodes. Similarly, according to our previous experiments and the study involving entomopathogenic and slug-parasitic nematodes, compost (green waste compost from Integraal Verantwoord Milieubeheer, vermicompost of PurVer NV, ‘Zöld Híd Komposzt’ green yard waste compost from Zöld Híd Régió Kft.) did not have any harmful effect on these beneficial nematode groups (Herren et al., 2018; Petrikovszki et al., 2019).

According to previous studies, every leaf litter and straw material sample contained certain amounts of tannic acid (Peng and Jay-Allemand, 1991; Gessner and Chauvet, 1994; Scutareanu and Lingeman, 1994; Anderson, 2005). In our experiment, straw and walnut leaf litter extracts had the lowest tannic acid contents (0.02 and 0.08%). In the case of straw, mortality was quite low (8.3%) at this tannic acid content, while walnut leaf litter extracts had total lethal effect (100%). It can be explained by other compounds of walnut leaves, for example juglone, which compound is characteristic to *Juglans* species and well-studied for its allelopathic effect (Mahajan et al., 1992; Cosmulescu et al., 2011). It seems quite clear that besides tannic acid, many other compounds in the examined leaf litters may have nematicidal effects, resulting total mortality at higher concentration levels.

Besides tannin, flavonoids, terpenoids, saponins, and alkaloids, phenolic and organosulfur compounds have different extents of nematostatic or nematicidal effect in plants (Chitwood, 1992; Desmedt et al., 2020). Phenolic compounds. like coumestrol, juglone, or m-coumeric acid have high (more than 95%) lethal effect on *M. incognita*. The efficacy of saligenin, quercetegetin or genistein is approximately 50%, and quercetin or fuslin have lower (around 20%) nematicidal activity (Mahajan et al., 1992).

Considering the fact that while leaf materials and straw contained tannic acid, this deterrent compound was not detected in compost extracts, it has been supposed that the lack of nematicidal effect of compost can also be attributed to biodegradation: if compost contained a compound with a nematicidal effect, it may have been degraded during composting. Zhang et al. (2020) proved that tannins and saponins from shell and seed cake of *Camellia oleifera* Abel can reduce during the composting process. They noticed that at the beginning of composting, tannins reduced the activity of certain thermophilic microbes. However, from the 20^th^ to 60^th^ days, microbes may adapt for tannins and the higher microbial activity accelerates organic matter decomposition, which produces heat, and tannins start to decompose. This is indirectly supported by the finding from Tirczka et al. (2015) regarding the effects of fresh walnut leaves and immature compost versus mature compost on white mustard (*Sinapis alba* L.) germination, emergence, and growth.

The results of the area choice tests are in agreement with the mortality test. Compost extract did not influence *M. incognita* juveniles. Significant repellent effects were observed in the presence of the examined leaf litter and straw extracts, however, this cannot be related to tannic acid content. In contrast, Hewlett et al. (1997) noted that tannic acid attracted *Meloidogyne arenaria* Neal and *M. incognita* juveniles. They used pure tannic acid for their experiments, which could have a stronger scent than our natural mulch-derived extracts, as these contain not only tannic acid but other compounds which could influence the preference of *M. incognita* juveniles.

Plant roots contain tannin, which serves as a signal for infective juveniles to find the root in the soil more easily, according to Ohri and Pannu (2010). For this reason, the repellent effect experienced was probably not caused by tannin, but by other substances. It is possible that the withered leaves we used in our experiments already emitted substances that indicate that the plant is no longer viable, which could cause repellence.

The nematicidal effect of various plant materials with high tannin content has already been proven. The effect experienced was found in reduced root damage, nematode population density, recovery and reproductive capacity, and immobility of juvenile individuals (Mian and Rodríguez-Kábana, 1982; Maistrello et al., 2010; d’Errico et al., 2018). However, there is little information about exactly what changes tannin induces in plant-parasitic nematodes.

The effects of tannin-rich plants on intestinal parasitic nematodes have already been investigated in ruminants (Thompson and Geary, 1995). Basically, the anthelminthic effect of tannin is attributed to two types of action mechanisms: indirectly, tannin strengthens the immune system of the host animal against parasites; in addition, it can directly affect the life activity of parasitic nematodes. In the case of direct effects, these can be divided into two types: changes in hypodermis, and neurophysiology or neuromuscular problems. The cuticle of nematodes is rich in proline and hydroxyproline. Besides the body surface, it can be found laced at the buccal cavity, esophagus, cloaca, and genital region (Thompson and Geary, 1995). Tannin can cause changes in the hypodermis and degradation in muscular and intestinal cells; furthermore, it can inhibit shedding and bind proteins (Hoste et al., 2006; Brunet et al., 2011; Tresia et al., 2016; Greiffer et al., 2022).

Glazer et al. (2015) investigated *Heterorhabditis bacteriophora* Poinar entomopathogenic nematodes found in African cotton leafworm (*Spodoptera littoralis* Boisduval), which consumed plants with high tannic content. In a previous experiment, Hoste et al. (2010) noted tannin aggregates around the buccal cavity of the intestinal parasite *Haemonchus contortus* (Rudolphi) Cobb. These findings were proved by Glazer et al. (2015) who also found that tannin inhibited recovery and development in the examined nematodes.

In addition, tannin causes nervous system and sensory problems (Tresia et al., 2016). This is confirmed by Engström et al. (2016), who noticed damage to the sensory organs around the buccal region of J1 and J2 stage juveniles, which could later lead to the death of the juveniles.

In the current experiments, there was no examination of cuticle of tested juveniles under electron microscope. Therefore, we cannot confirm the hypothesis that hypodermis was damaged by tannic acid. However, during the evaluation of the mortality test, infusion of 5% of lactic acid was performed to confirm whether the individuals had actually died. Despite the fact that they appeared to be immobile, lactic acid made them move if they were alive. Otherwise, if they were dead, they did not move at all. From this, it can be concluded that tannic acid may have affected their muscular and/or nervous system, as a result of which they were no longer able to move.

There are differing opinions on how pH affects juveniles of plant-parasitic nematodes. Wang et al. (2009) suggests that pH between 4.5 and 5.4 has an effect of attraction on juveniles of *Meloidogyne* species. In contrast, according to Rocha et al. (2017), alkaline pH value (8) attracts, while acidic pH (5) repels *M. incognita* juveniles. Despite the fact that pH can also indicate the position of the plant roots in the soil (Nichol and Silk, 2001), the relationship between area choice and pH did not show a close relationship.

Current and similar experiments facilitate and contribute to better understanding of nematological aspects and the mechanisms of soil surface mulching. There are still several research directions to be developed: observation of egg hatching, mobility of juveniles, inclusion of additional compounds of mulch materials and species to be tested not only in plant-parasitic but also in non-target nematodes and other soil-dwelling organisms in order to contribute to the development of more environmentally friendly plant protection strategies.
